# I am not autonomous enough! The role of autonomy beliefs in the relation between social stigma and recovery

**DOI:** 10.1192/j.eurpsy.2022.1521

**Published:** 2022-09-01

**Authors:** D. Lampropoulos, H. Klaas, D. Spini

**Affiliations:** 1 University of Lausanne, Swiss National Centre Of Competence In Research Lives, Switzerland, Lausanne, Switzerland; 2 FORS, Fors, Lausanne, Switzerland

**Keywords:** stigma, Liberal values, self-stigma, Recovery

## Abstract

**Introduction:**

It has been suggested that liberal values such as lack of autonomy and burden discourses shape the public’s relation toward people with health problems. However, previous research on the role of such values on one’s recovery and well-being is scarce.

**Objectives:**

We investigated whether perceived autonomy mediates the impact of stigma and negative social experiences on life satisfaction and recovery.

**Methods:**

Our sample, drawn from a subsample of the Swiss Household Panel survey, consisted of 326 individuals reporting a mental health problem (50.3% women, Mage = 50.7, SD = 13) and 354 individuals reporting a physical health issue (49.7% women, Mage = 53.6, SD = 14.7). We tested a model where perceived autonomy, measured with four items drawn from the Acceptance of Illness Scale (AIS), mediates the impact of experienced stigma and negative social experiences on health satisfaction and self-reported recovery.

**Results:**

Our analysis of direct and indirect paths confirmed our hypothesis. Our model showed a good fit to the data for persons with a mental health problem (CFI = .984; RMSEA = .038) and an adequate fit for persons with a physical health problem (CFI = .92; RMSEA = .080).

**Conclusions:**

Our results provide empirical evidence for the potentially self-stigmatizing role of the autonomy ideal for people with health problems and invite for the development of further research and practice regarding this role.

**Disclosure:**

No significant relationships.

